# Anxiety of Older Persons Living Alone in the Community

**DOI:** 10.3390/healthcare8030287

**Published:** 2020-08-22

**Authors:** Jinna Yu, Kwisoon Choe, Youngmi Kang

**Affiliations:** 1 The Graduate School, Chung-Ang University, 84 Heukseok-ro, Dongjak-gu, Seoul 06974, Korea; dbwlssk79@naver.com; 2 Department of Nursing, Chung-Ang University, 84 Heukseok-ro, Dongjak-gu, Seoul 06974, Korea; 3 East-West Nursing Research Institute, College of Nursing Science, Kyung Hee University, 26 Kyungheedae-ro, Dongdaemun-gu, Seoul 02447, Korea; ykang@khu.ac.kr

**Keywords:** anxiety, living alone, older persons, qualitative research

## Abstract

Anxiety is a common mental health problem among older persons, and the prevalence is higher in those who live alone than those who live with others. This study aimed to explore the experiences of anxiety in older persons living alone. A descriptive phenomenological approach was used to collect and analyze the interview data from 15 older persons (5 males, 10 females) living alone in Seoul, South Korea. Four main themes emerged from the data analysis: fear of being alone, concern about having an aged body, apprehension mixed with depression and loneliness, and fear of economic difficulties. These findings indicate that older persons living alone should receive continuous attention to prevent them from being neglected and their anxiety from worsening. Above all, it is vital to ensure comprehensive support for older persons living alone to alleviate their anxiety.

## 1. Introduction

The number of older persons aged 65 years or older living alone is expected to steadily increase globally [1,2,3]. Approximately 28% (14.3 million) of American older persons lived alone [1]. In South Korea, the proportion of one-person households aged 65 years or older among all households increased from 3.8% in 2000 to 7.2% in 2018 [4].

Older persons living alone experience mental health problems such as anxiety, fear, loneliness, isolation, and feelings of uselessness due to aging [5]. They experienced greater psychological distress such as feelings of hopelessness, nervousness, restlessness, sadness, and worthlessness compared to those living with spouses [6] as well as depression, and loneliness [7]. Above all, anxiety is one of the most common mental health problems of older persons [8].

Anxiety is defined as an emotional state characterized by tension or anxious thoughts [9]. Although anxiety is a normal part of life, and possibly a driving force [10], a high level of anxiety among older persons is associated with various illnesses such as cardio-cerebrovascular problems [11,12,13,14], decreased cognitive function [15,16], sleep disturbance [17,18], and unhealthy behaviors such as smoking and drinking alcohol [19]. Older persons with high anxiety may exhibit social restrictions, so they stay at home or interact only with close neighbors [20].

The authors wondered how older persons living alone experience anxiety. As a result of the literature review, one qualitative study [21] was identified on the death anxiety of older persons living alone. This qualitative research revealed that older persons, who live alone, were afraid of losing independence rather than death itself, and older persons were worried that someone would or would not find the body after dying alone at home. The exploration of anxiety in older persons living alone has not been sufficiently conducted. Therefore, this study aimed to explore the anxiety experienced by older persons living alone using a phenomenological approach.

## 2. Materials and Methods

### 2.1. Participants

Participants were recruited via snowball sampling at one senior welfare center in Seoul, South Korea. The senior welfare center provides daytime recreational programs for senior citizens living in the community. The inclusion criteria for participants were those aged 60 years or older, living alone, and being able to communicate without a diagnosis of cognitive impairment, such as dementia.

Fifteen individuals (10 females and 5 males; mean age 79.3 years; range 63–87 years) participated in this study (Table 1). All participants were either unmarried, divorced, or bereaved, and were living alone (despite having adult children). Participants were asked regarding any diagnosed diseases to identify whether the disease state influenced their anxiety, and it was found that participants had diseases such as hypertension, diabetes, heart disease, herniated intervertebral disc, and arthritis, as well as mental illnesses such as depression and alcoholism.

### 2.2. Ethical Considerations

This study was approved by a university institutional review board (1041078-201612-ZZ-220-01). All participants were informed about the purpose and procedure of this study and completed a written consent form.

### 2.3. Data Collection

Data were collected from four group interviews (13 participants) and two individual interviews (2 participants). First, qualitative data were collected through face-to-face group interviews and additional interviews with two individuals as they requested individual interviews. Most participants preferred group interviews, which involved participants who were close to each other and wanted to share their experiences. The interviews were conducted in a counseling room at the senior welfare center. The average interview time was 1 h (range: from 50 to 1 h 20 min). All interviews were audio-recorded with participants’ consent and transcribed by an author. Follow-up individual interviews were conducted with 8 of the 15 participants to resolve ambiguities and to ensure the accuracy of the transcriptions.

During the interviews, participants were asked to describe situations where they felt anxious when living alone, using the following questions: “Have you experienced anxiety? If so, describe the situations.” “What did you do when you felt anxious?” “What mitigated or worsened the anxiety?” “What differs between when you feel anxious and when you do not?” “Have you ever sought out a therapist or an expert to manage your anxiety?” Interviews were held until data saturation was achieved—that is, the point at which no new conceptual aspects, nuances, or perceptions of anxiety emerged [22].

### 2.4. Data Analysis

Data were analyzed using a descriptive phenomenological approach. The audio-recorded interview data were transcribed verbatim by one of the authors. First, the authors read the transcripts several times individually to obtain the text′s overall meaning. Second, the authors demarcated the text and underlined the sentences or phrases focusing on the participants′ anxiety. Third, they summarized the meaning of each sentence and made a coding list together. All authors met several times to review the coding list and to discuss how to categorize them into sub-themes and themes. An example of this step is presented in Table 2. In the data analysis, all first-person expressions were changed into third-person expressions to maintain a clear boundary between the participants′ perspectives and the authors′ own [23].

## 3. Results

Four main themes were extracted: “fear of being alone,” “concern about having an aged body,” “apprehension mixed with depression and loneliness,” and “fear of economic difficulties.”

### 3.1. Fear of Being Alone

Participants feared being alone and found living alone to be lonely, empty, and anxiety provoking. They expressed feeling desolate when entering a dark house without anyone waiting for them and anxious and empty when going to bed. *“Sometimes, I get around and I go home late. When I go home and open the door, there is no one there. It′s a bit lonely because there is no one to wait”* (Participant OS, female, age 87). When participants were alone at home, they thought about their past and worried about the future, and often felt that they wanted to die quickly because they did not enjoy life. Some participants lacked friends nearby and felt that their lives were too miserable and that it was too painful and lonely to live alone.

Some participants had no children, whereas others had children but were not in contact with them. In both cases, participants felt anxious and nervous because they had no one to depend on. During holidays, they felt the loneliest because they knew that while others their age were with their families, they were alone. Participants, whose spouse had died, felt sad that their children did not visit them. Furthermore, they sometimes resented God or the spouse that died before them.

“Then [when I attempted suicide], I resented my wife. [To wife] Are you comfortable going there alone? I have a house, I have money, but I do not need everything. Is this happiness to me? If there is a God, God is too bad”.

(Participant SG, male, age 76)

When alone in the evening, participants sometimes became afraid of the sounds outside their home. Night-time anxiety also produced restlessness and a feeling of not knowing what to do. Such feelings made participants seek out others: on weekdays, they went to senior welfare centers or the older persons’ shelter at their community service center, whereas on weekends, they spent time in parks and markets, listening to people—even strangers—talk.

“I’m never at home. If I′m at home, I′m bored. But if I go outside, it’s okay. I just go around the park. There are strangers in the park. I do not know anyone there but I sit alone among these strangers, listening to their stories and spending time, and then come home”.

(Participant YS, female, age 76)

Participants also turned on the TV to eased their loneliness, both during the day and at night. They sometimes sang songs at home or exercised outside, and received some comfort from their religion. *“I rely on God. It is very important for me to depend on someone. But I have no one to depend on and I cannot depend on anyone. People need to depend on something and people are anxious if they cannot depend on something. So, I am a little comforted because I depend on God”* (Participant YG, male, age 85).

Participants felt anxious about dying alone and though a lot about death when they slept alone at night. Most participants expressed wanting to die comfortably at night, or to die suddenly, without pain, in an accident. Participants also worried that their bodies would be discovered long after their deaths. It produced more anxiety than did death itself, given their lack of social contact. *“I am not afraid to die, but I worry about who will clean up my body if I die”* (Participant SH, male, age 78).

### 3.2. Concern about Having an Aged Body

Older persons were concerned about their aged and often painful bodies. All participants except one had a chronic disease such as hypertension, diabetes mellitus, and arthritis. They said that they worried about becoming sick when alone. While they felt it natural for them to die, given their age, they hoped their death would be painless and they would die well. They felt it was sad and more anxiety-provoking to be sick than to die because if they were sick alone, they would have no one to accompany them to the hospital or to care for them when bedridden.

“Anxious? I’m the most anxious when I’m sick because I’m alone and sometimes I just feel anxious and my mind is not stable. I do not have anyone to care for me when I am sick”.

(Participant KS, female, age 82)

Some participants did not want to look shabby when feeling sick or hear from others that their bodies smelled. They kept themselves clean and dressed up: *“If I am shabby because I am sick, others will look down on me. I would be too sad to hear from others that I smell. No matter how hard it is, I have to dress up each day”* (Participant OJ, female, age 77). As participants aged, they constantly became ill. *“I get sick because I get older, so I often feel sad, because I cannot move my body freely”* (Participant KS, female, age 82). Anxiety made participants feel sicker and weaker. *“My whole body shakes like [I have] Parkinson’s disease. I don’t feel this when I work during the day, but my body shakes when I lay down at home”* (Participant DS, male, age 63). *“The pain is worse at night. That′s so strange. I feel worse at night”* (Participant SH, male, age 78).

Many participants suffered from insomnia. Some participants felt that they would die because of their insomnia. “I have suffered a lot because of my insomnia. I did not think I was nervous, but I could not sleep … I was really troubled. Then I felt that I could die like this” (Participant ZJ, female, age 78). Additionally, participants often had disturbing dreams, which made them feel strange and lead to dark thoughts about death that made it difficult for them to sleep through the night.

### 3.3. Apprehension Mixed with Depression and Loneliness

Participants′ anxious feelings were mixed with depression and loneliness, and participants experienced these three feelings simultaneously as one negative emotion. *“I am lonely, anxious, and nervous when I am at home alone in the evening. Then I get the feeling that I′m depressed”* (Participant HS, female, age 83). Their feelings of anxiety and depression often arose from thoughts about their past, particularly times of struggle. Many sighed constantly, expressed anxious thoughts, and even cried as they talked about their past: *“I sigh a lot. I know that sighing is bad but when I sigh, my mind stabilizes”* (Participant YS, female, age 76).

Some participants felt that they unnecessarily hated others or wanted to physically harm them. “I′m just anxious, and I want to fight with others without a reason. When I′m nervous, it′s worse. However, sometimes I feel uneasy and sometimes I feel scared. Sometimes [though] I feel anxious and I want to fight with someone” (Participant DS, male, age 63). They expressed that they would rather die than live without any fun, and felt bitter about living alone. Three male participants had a history of suicide attempts through overdosing on sleeping pills or slashing their wrists. “I tried to commit suicide three or four years ago. I thought it would be painful, so I drank two bottles of alcohol and then I tied my wrists and slit my wrist arteries twice with a razor blade” (Participant SG, male, age 79).

### 3.4. Fear of Economic Difficulties

Most participants felt worried about living without money. They said that they worried about their health first, then about money. Most participants were able to meet their expenses through government support; however, older persons who did not receive such support experienced difficulties because of a lack of living expenses. *“I have been the most anxious since the government’s support stopped. I am ashamed to live in poverty. My son also has limited resources, so I want to die. However, I live because I cannot die”* (Participant YS, age 76). Some participants worried about having to move out of their house when they had no money. They felt that they could not continue paying monthly rent because the prices kept rising. *“House prices are rising every year, but it is difficult to keep paying monthly rent. Nowadays, I cannot sleep because of it. I am more concerned about my house. How can I leave my house?”* (Participant SH, male, age 78).

Participants were unable to pay for any hobbies and had to remain at home on weekends. They said that nowadays, everyone, even an older person, needs money wherever they go, including going out and playing. Even at church, those who have money receive different treatment. *“I do not go to church. I do not have anything. I have to give offertories to the church if I go. So I do not go anywhere. Where is the free money in the world? If someone gives an offertory to the church, I have to give one too”* (Participant YS, female, age 76). *“If I do not give money in church, others ignore me. People discriminate against those who do not have money. This is a rule. The church is the same”* (Participant JN, female, age 83).

## 4. Discussion

This study explored how older persons living alone experienced anxiety in the community. All participants in this study expressed a fear of being alone. Furthermore, most participants were anxious and felt no joy in living because they were alone. Some participants expressed their fears of being alone, in particular, when talking about death. For these older persons, the thought of death was combined with worries that their bodies would not be discovered until long after their death. This finding was consistent with those of Caswell and O’Connor [21], which found that older persons living alone were afraid that others, including their family, would only discover their bodies long after their death. Many older persons living alone in this study seemed more afraid of their bodies going undiscovered than death itself. This contrasts with one study of older persons living in care institutions, whose fear of death itself was exceedingly high [24]. Therefore, to reduce fears of dying alone, it is necessary for older persons living alone to receive visits from nurses or social workers, and simultaneously make friends in their communities. Another way of alleviating anxiety about dying alone is to install devices such as emergency call bells connected to the public safety call center at the bedside of older persons, allowing them to call for help during emergencies quickly.

Participants’ anxiety also stemmed from their aged bodies. Older persons experience a progressively higher risk of multiple chronic diseases with age [25], making it difficult for them to live independently [21]. All participants had chronic diseases such as arthritis and high blood pressure and experienced a vicious cycle of feeling sick when anxious and more anxious when they were sick. This accords with a previous study on older persons with osteoarthritis, which showed that older persons who were initially anxious were more likely to report pain after three years. Conversely, older persons who initially had pain were more likely to have anxiety after three years [26].

Furthermore, older persons with poorer physical health were more likely to be afraid of death [24], and older persons who have a poor subjective health status had a 2.3-fold higher risk of experiencing anxiety than do older persons with good subjective health status [27]. For example, older persons with hearing loss or those who take more than four medications because of physical illness are likely to develop anxiety disorders [28]. In addition, older persons who are physically frail (i.e., exhibit decreased walking speed, impaired grip, decreased physical activity, greater subjective fatigue, and weight loss) are more anxious than robust older persons [29]. These results suggest that initial screening and intervention for physical illnesses that exacerbate anxiety in older persons living alone are necessary.

In this study, the experiences of anxiety in older persons were complex, usually mixed with loneliness and depression. Similarly, older persons living alone experienced multiple psychological problems, such as, anxiety, fear, loneliness, isolation, and feelings of uselessness due to aging [5]. Older persons with chronic physical illness experienced feelings of anxiety, restlessness, and sadness when they talked about feelings of loneliness [30]. Older persons who felt lonely exhibited a higher prevalence of anxiety than older persons who did not feel lonely [31].

In this study, older persons also complained of depression when talking about their anxiety. Depression and anxiety often co-occur—in the Swedish general population, the comorbidity rate was approximately 50% [32]. Moreover, older persons who experience anxiety are more likely to experience depression [27]. Older persons with anxiety had an 8.7-fold higher risk of suicidal thoughts than older persons without anxiety, indicating that anxiety can instigate or exacerbate suicidal ideation in older persons [33]. Regarding anxiety, depression, and loneliness, anxiety increases depression and simultaneously mediates the relationship between loneliness and depression [31].

Therefore, to reduce anxiety among older persons living alone, it is necessary to identify loneliness and depression as well as anxiety. Moreover, establishing self-help groups among older persons with similar mental health problems and a system whereby older persons can receive continuous counseling and treatment for such problems are also helpful.

Finally, a fear of economic difficulties made older persons in this study anxious. Most participants were completely dependent on government subsidies. In South Korea, the poverty rate among older persons aged 65 years or over is nearly 45.7%, which is the highest among the Organization for Economic Co-operation and Development (OECD) countries [34]. For comparison, the poverty rate among older persons in the United States was 20.9%, meaning that one in five older persons faces poverty [34]. Older persons living alone were less likely to own their own house and have less income per household than older persons living with families [35]. Studies have found that economic stress is high among older persons living alone, which not only lowers their quality of life but also negatively affects their mental well-being [36]. Stress associated with economic problems was found to increase anxiety among all age groups, including older persons, in low-income families in the United States [37]. In this study, older persons also expressed worries about medical expenses and the need to visit the hospital because of their economic difficulties, which aligns with another study demonstrating that financial problems related to medical expenses were a major source of anxiety among older persons in Portugal [38].

Based on these results, it is pivotal to reduce the economic burden of older persons living alone, perhaps by selecting and supporting those individuals in need by accurately measuring their financial status. Reducing housing-related burden by expanding residential spaces such as shared living homes for older persons is also essential. Providing older adults with jobs that consider their functional status and government subsidies may be helpful, as this would allow them to cover their costs of living.

Our findings are meaningful because they enhance the understanding of anxiety as experienced by older persons living alone. Nevertheless, this study has several limitations. All participants were associated with a senior welfare center in one city in South Korea. Therefore, this study could not explore the anxiety experiences of older persons living alone who lacked any social support system, or older persons from different geographic areas. Besides, to understand older persons’ anxiety better, it is necessary to study the anxiety of older persons who live with their family members or without financial difficulties and compare the results of the studies with those of this current study.

## 5. Conclusions

The findings of this study revealed the anxiety experienced by older persons living alone. Health professionals working in the community should seek measures to reduce older persons’ fear of being alone, aged body, depression and loneliness, and financial difficulties to alleviate their anxiety. This study also provides a theoretical rationale for the early detection and management of anxiety in older persons living alone in the community.

## Figures and Tables

**Table 1:** Demographic characteristics of participants.

Group /Individual Interview	Participants	Sex	Age	Marital Status	Diseases
Group 1	HS	Female	83	Bereaved	Hypertension, Herniated intervertebral disc, Arthritis
	JN	Female	83	Divorced	Hypertension, Diabetes mellitus, Arthritis, Cancer
	YS	Female	76	Bereaved	Herniated intervertebral disc, Arthritis
	WS	Female	85	Bereaved	Hypertension, Diabetes mellitus
Group 2	SH	Male	78	Bereaved	Hypertension, Heart disease, Hyperlipidemia, Herniated intervertebral disc
	DS	Male	63	Divorced	Hypertension, Heart disease, Alcoholism
	HJ	Male	76	Unmarried	Hypertension, Stroke
Group 3	YJ	Female	74	Divorced	Hypertension, Gastritis, Herniated intervertebral disc
	KS	Female	82	Bereaved	Herniated intervertebral disc, Femur fracture
	OJ	Female	77	Unmarried	Arrhythmia, Herniated intervertebral disc
Group 4	ZJ	Female	78	Divorced	Hypertension, Osteoporosis, Depression
	OS	Female	87	Bereaved	Hypothyroidism, Arthritis, Hyperlipidemia, Depression
	MO	Female	87	Bereaved	Hypertension, Diabetes mellitus, Arthritis
Individual interview	YG	Male	85	Bereaved	None
	SG	Male	76	Bereaved	Rheumatoid arthritis

**Table 2:** An example of data analysis to obtain subthemes and themes.

Meaning Unit	Transformation	Subtheme	Theme
Participant 5 worries more about whether his body will be rotten at home, or who will take care of his body after dying in a house without anyone rather than being afraid to die.	Participants reported that they were more worried about the situation after their death than they were afraid to die because they lived alone, and did not know what will happen after they die. They are afraid that their body will rot at home, or the body would not be taken care of after their death.	Worry about dying alone and after-death situation.	Fear of being alone
